# Re-emerging Lassa fever outbreaks in Nigeria: Re-enforcing “One Health” community surveillance and emergency response practice

**DOI:** 10.1186/s40249-018-0421-8

**Published:** 2018-04-28

**Authors:** Ernest Tambo, Oluwasegun T. Adetunde, Oluwasogo A. Olalubi

**Affiliations:** 1 Africa Disease Intelligence and Surveillance, Communication and Response (Afric DISCoR) Institute, Yaoundé, Cameroon; 2 Higher Institute of Health Sciences, Universite des Montagnes, Bangangte, Cameroon; 3 Department of Geography and Environmental Management, University of Ilorin, Ilorin, Kwara State Nigeria; 4 Department of Public Health, Kwara State University, Malete, Kwara State Nigeria

**Keywords:** Evidence, Early warning, “One Health” approach, Surveillance, Lassa fever, Response, Nigeria, Africa

## Abstract

We evaluated the impact of man-made conflict events and climate change impact in guiding evidence-based community “One Health” epidemiology and emergency response practice against re-/emerging epidemics. Increasing evidence of emerging and re-emerging zoonotic diseases including recent Lassa fever outbreaks in almost 20 states in Nigeria led to 101 deaths and 175 suspected and confirmed cases since August 2015. Of the 75 laboratory confirmed cases, 90 deaths occurred representing 120% laboratory-confirmed case fatality. The outbreak has been imported into neighbouring country such as Benin, where 23 deaths out of 68 cases has also been reported. This study assesses the current trends in re-emerging Lassa fever outbreak in understanding spatio-geographical reservoir(s), risk factors pattern and Lassa virus incidence mapping, inherent gaps and raising challenges in health systems. It is shown that Lassa fever peak endemicity incidence and prevalence overlap the dry season (within January to March) and reduced during the wet season (of May to November) annually in Sierra Leone, Senegal to Eastern Nigeria. We documented a scarcity of consistent data on rodent (reservoirs)-linked Lassa fever outbreak, weak culturally and socio-behavioural effective prevention and control measures integration, weak or limited community knowledge and awareness to inadequate preparedness capacity and access to affordable case management in affected countries. Hence, robust sub/regional leadership commitment and investment in Lassa fever is urgently needed in building integrated and effective community “One Health” surveillance and rapid response approach practice coupled with pest management and phytosanitation measures against Lassa fever epidemic. This offers new opportunities in understanding human-animal interactions in strengthening Lassa fever outbreak early detection and surveillance, warning alerts and rapid response implementation in vulnerable settings. Leveraging on Africa CDC centre, advances in cloud-sourcing and social media tools and solutions is core in developing and integrating evidence-based and timely risk communication, and reporting systems in improving contextual community-based immunization and control decision making policy to effectively defeat Lassa fever outbreak and other emerging pandemics public health emergencies in Africa and worldwide.

**Electronic supplementary material:**

The online version of this article (10.1186/s40249-018-0421-8) contains supplementary material, which is available to authorized users.

## Multilingual abstracts

Please see Additional file 1 for translations of the abstract into the five official working languages of the United Nations.

## Background

The increasing evidence of climate change and other man-made conflict events impacts on emerging and re-emerging zoonotic or vector-borne diseases such as Lassa fever outbreak direct risk-effect occurrence and burden worldwide and particularly in developing countries. Acute Haemorrhagic Fever Syndrome is a general term broadly attributable to diverse mild to severe group of animal and human illnesses that encompass: Lassa fever (arenaviridae), Rift Valley fever (RVF), Crimean-Congo haemorrhagic fever (CCHF) (bunyaviridae), yellow fever (flaviviridae), Ebola and Marburg viral diseases (filoviridae), dengue (dengue haemorrhagic fever (DHF) and other viral diseases such as rickettsial or bacterial diseases with ability to result in epidemics [1, 2].

Lassa fever a known endemic infectious disease of poverty has emerged as a severe outbreak of public health threat and burden in Nigeria in the recent past [1]. Nigeria is currently experiencing a smouldering Lassa fever outbreak in almost 20 states, 175 confirmed and suspected cases and 101 deaths since August 2015 have been reported. The outbreak has been imported into neighbouring country Benin, where 68 cases and 23 deaths have been profiled. Interestingly, the death rate in 70% of the current outbreak (83 laboratory confirmed cases died) is much higher than previously documented and the reasons are still unclear [3, 4]. Sequel to the successful containment of Ebola outbreak by the Nigerian government in 2014 and the wider appraisal by the international community, recent Lassa outbreak reveals some inherent gaps and raising health system challenges in determining how Nigerian communities and other prone countries can proactively mitigate, prepare and respond to this emerging and re-emerging infectious disease of poverty. The Lassa virus is transmitted by rodents and poses potential disease ecology and public health impact [3–5]. The first case of Lassa fever globally was identified in Lassa, a settlement in Borno State, North East Nigeria in 1969 [6, 7]. It is spread by contact with infected rodent’s feces or urine, inhaling contaminated dust, eating contaminated food or by contact with the fluids of an infected person dead or alive [2, 5, 8, 9]. The multimammate mouse, *Mastomys natalensis* is the rodent reservoir of the *Arena spp*. the virus responsible [10]. Following contact tracing, 80 percent of those infected remain asymptomatic while acute syndromic manifestations include fever, weakness, nausea, vomiting and diarrhoea leading to 1–15% severe cases of bleeding coma and death [2, 3]. Spatial and epidemiological mapping of vulnerability coupled with laboratory biomarkers (immunoglobulin M (IgM) antibody) or related molecular assays are useful tools in early detection, virus isolation and confirmation of positive case [1, 2].

Nigeria is no doubt now endemic for Lassa fever, there was an observed 21.3% seropositive prevalence in a countrywide study [5]. A brief comparison between January to August for 2016 and 2017 was made for Lassa fever virus burden in Nigeria. In 2016 by 32^nd^ week, 9.53% of suspected cases were confirmed by laboratory tests. However, of the 75 laboratory confirmed cases 90 deaths occurred i.e. 120% laboratory case fatality. That means 20% of observed Lassa fever related deaths were not confirmed as cases by laboratory tests hinting to a systems gap in the disease detection and surveillance. However, by 2017, this observed health systems gap in infectious disease and outbreak detection and surveillance was not appropriately addressed [4]. Of the suspected Lassa fever cases, 24.68% were laboratory confirmed while 59.79% of laboratory confirmed cases ended up in deaths. This showed a remarkable improvement against the previous year when mortality was experienced outside of laboratory confirmed cases. In this scenarios, 40.21% of laboratory confirmed cases has been helpful to improve case treatment and reduced Lassa fever morbidity and mortality. A comparison between s 2015 and 2016 Lassa fever epidemics showed how overwhelmed the health system in Nigeria was at that time. It was observed that 5.81% of suspected cases were laboratory confirmed. However, 16.0% of laboratory confirmed cases resulted in deaths. That means 60% of Lassa fever related deaths were not accounted for by laboratory confirmation. In year 2016, 11.83% of suspected cases were confirmed in the laboratory. Just like the previous year though with a lower margin, 109% of laboratory confirmed cases resulted in deaths. Thus 9% of Lassa fever related deaths were not accounted for through laboratory tests to confirm such cases.

The paper assesses the current trends in re-emerging Lassa fever geo-spatial distribution, inherent gaps and raising health system challenges towards improving interlinkage of laboratory and epidemiology surveillance to evidence for community towards one health approach and practice.

### Building trans-disciplinary, human, veterinary and phytosanitation preventive and control measures

Since the Lassa fever virus is transmitted to humans via contact with food or household items contaminated by rodent hosts, sexually or direct/indirect contact with body fluids such as the blood, urine, and saliva of an infected person [2, 5], an integrated “One Health” (animal-human-environment) approach is the best fit-for-purpose to mitigate this re-emerging Lassa fever epidemic scourge in Nigeria and beyond. A study of 18 different areas in Guinea, a West African country confirmed that only the *M. natalensis* and not *M. erythroleucus* correlates geographically with observed Lassa fever seropositivity prevalence in humans [10]. A study carried out in rural endemic Esan West local government area in Southern Nigeria showed seroprevalence at 58.2% where 96.1% of houses had seen rodents in the previous 6 months [11], similar to 24 cases reported in Ondo state in January 2018. It was further observed that there was no much focus on rodent control measures in public awareness within the study area calling out the need for further studies to develop culturally effective and acceptable design measures and capacity development which are affordable in dealing with the infectious rodents [11]. Peak incidence of Lassa fever in Sierra Leone has shown to overlap the dry season from the wet season falling between May to November annually [12]. However, peak season has been known to occur in the dry season between January to March [13]. The endemic swath area from Senegal to Nigeria fringes make up the hotbed for Lassa fever virus outbreak preparedness and response backed with active surveillance team close follow up, active case searching and contact tracing, laboratory support and disease awareness in West Africa [10, 14, 15].

Hence, in absence of preventive medication or vaccine against Lassa fever, increasing community awareness and health education to avoid contact with reservoir sources mainly rats, prevention of food infestation rodent’s and food safety practice to appropriate waste management coupled with improved water, sanitation and hygiene (WASH) program implementation is crucial. Since, sexual transmission of Lassa virus has also been reported, improved access to sexual and reproductive preventive measures is also important in line with WHO recommendations as well as shared traveller information support.

Importantly, there is an urgent need to linking disease ecology with enhanced surveillance data garnered from 1969 across Sub-Saharan Africa [6]. However, this attempt was faced with the challenge of relying on probabilistic models for mapping [6] due to the temporal and spatial scale of the work as well as paucity or absence of comprehensive and coherent infectious diseases emergence and spread or burden backed with robust data sharing and risk communication across multidisciplinary and intersectorial fields. There is an urgent need to concisely map the risk factors (national, regional and geographic socio-cultural/socio-demographic/socio-economic variables) and seroprevalence, reservoirs and case mortality interaction [12, 16].

### Strengthening Lassa fever epidemiologic risks surveillance and early laboratory detection

Strengthening local and regional robust and sustainable integrated disease surveillance and response (ISDR) implementation into routine laboratory diagnostic and epidemiologic surveillance services, and surge resource capabilities is imperative. Scaling up adequate community social mobilization, nationwide enlightenment and health education outreach coverage using various social media and mass media outlets by various stakeholders is needed to prevent and respond promptly to potential epidemic events at each identified community health system level [1]. Leveraging on decentralized Africa Centers for Diseases Control and Prevention and regional public laboratory network is crucial in promoting the WHO recommendations in global outbreak threat and crisis emergency response, advancing international health regulation (IHR), 2005 and global health security. Moreover, investing into more sensitive and reliable Lassa fever virus point of care and field diagnostic tools for early detection (eg. Rapid diagnostic kit) and rapid molecular case confirmation, safe and effective drug and vaccines is core in remote rural settings where vulnerable communities dwell with rodent’s reservoirs. Developing and integration effective and culturally-fit data and information sharing platform for improved awareness and risk communication strategies coupled with local outbreak surge capacities is necessary in strengthening health systems, emerging pandemics surveillance and emergency response interventions across Africa.

Leveraging on digital, cloud-sourcing and social media in developing and integrating timely risk communication and reporting systems can be seen to start from the lowest administrative level at the community up to Central or Federal level while a feedback process flows from the development partners and Federal government back to the communities. Fostering key operational coordination, epidemiology and surveillance capacity building programs should ensure effective and concurrent trans-disciplinary outbreak response actions and Lassa fever clinical case management guidelines. Hence, strengthening community health centres and laboratory capacity, data sharing access and operational logistics for evidence operational research priorities and decision making policies, supply chain and timely risk communication and share livelihoods [17].

### Integrating community-based “One Health” surveillance and emergency response practice against emerging pandemics

The Federal Government of Nigeria has embarked on integrated infectious diseases prevention and control; however integration in primary healthcare is still seldom and unstructured at all levels nationwide. Integrating community-based “One Health” surveillance and emergency response practice is crucial in addressing the persistent scourge of poverty-related Lassa fever outbreak and other emerging zoonotic disease pandemic threats amongst vulnerable populations across the region. In reviewing the 2016/2017 Lassa fever year, stakeholders hosted an interdisciplinary action review meeting in Abuja between 21^st^ and 22^nd^ of August, 2017, with the whole essence of building and strengthening robust and effective health system to achieve increasing access to universal health coverage (UHC) and sustainable development goals(SDGs). Noteworthy, was the technical support from the WHO Africa Regional Office (AFRO) and the National Lassa fever Steering Committee at the forum. The Nigeria Centre for Disease Control (NCDC) in partnership with the aforementioned as well as other supporting partners (e.g.: Diagnostic laboratories, Lassa fever treatment health facilities, laboratory and health emergency response teams, Federal Ministry of Environment, Federal Ministry of Agriculture and Rural Development) participants from State governments affected by Lassa fever endemicity and epidemics.

Scaling up contextually diagnostic and care access at the point of need no matter the location and time without any encumbrances is critical to improving vulnerable population quality of life, productivity, reduction financial impoverishment and poverty alleviation [18, 19]. The health system goes beyond just health facilities and medical personnel. It consists of numerous stakeholders (people, organizations and activities) working in concerted efforts with paramount intentions of maintaining, restoring and improving health across both individuals and groups [20]. Important components as government, leadership and funding is needed to improve medicines access and logistics, human resource capacity for healthcare service delivery and information technology uptake in health system strengthening [20, 21]. Thus, “One Health” Lassa fever epidemic surveillance and control offers new opportunities in understanding human-animal and environment interface and expanding zoonotic diseases public awareness, community preparedness and resilience strategies, and mitigation measures. Exploring emergency outbreak or disaster crisis insurance schemes initiative, immunization and medicine access, and integrated environmental/community health, veterinary to health professionals’ capacity development at all levels. This is essential to achieving optimum resource allocation, technical assistance and surge workforce deployment to mitigate the scourge of Lassa fever and future emerging outbreaks in Nigeria and across Africa.

Contemporary increasing in consumer/provider-generated mhealth technology and application, social media penetration and acceptance, online disease data and information literacy and communication to emergency response or recovery to immunization scale coverage and effectiveness should exploited to optimize health benefits, wellbeing impacts and return of investment as coupled with traditional mass media [22] in reaching out to people especially with the high penetration of mobile phone technology in the country. There is a need to apply appropriate effective risk communication strategies in improving disseminating and public health messages uptake, while increasing community health systems programs participation and resiliency for impact against emerging and re-emerging pandemics and epidemics threats [23–25].

### Building Lassa fever and other emerging Zoonotic diseases outbreaks early warning indicators and rapid response

In controlling the scourge of Lassa fever an early warning system and rapid response is important. Once one case of Lassa fever is suspected an alert should be made and once this is confirmed in the laboratory then the situation must be treated as an epidemic [1, 2]. This necessitates immediate further actions on the confirmed epidemic. The usual first point of call of the sick especially the terminally ill is usually the health facility. This puts workers in health facilities at greatest risk in contributing to outbreaks of Lassa fever. This is referred to as nosocomial transmission, infections acquired in the hospital either from patient to medical personnel, medical personnel to patient or patient to patient. As such, early and rapid diagnosis of suspected Lassa fever cases while adhering to standard operating procedures (SOPs) will help salvage such events [26]. Personal protection equipment and other standard dress codes, ward isolation, etc. should be strictly adhered to as a preventive measure [27]. It has been observed that nosocomial pathway has been a huge player in the spread of Lassa fever in Nigeria [28] showed how Lassa fever spreads through nosocomial transmission. As explained [29] the viral haemorrhagic fever (VHF) or Lassa fever patient gets into the hospital in Kwara, Bauchi, Ebonyi, Edo, Enugu, Kano, Kogi, Nasarawa, Ogun, Ondo, Plateau, Rivers, and Taraba states in Nigeria. Then, such a patient is attended to by both medical and non-medical personnel, patients and visitors at the hospital are put at risk. Viral transmission via unprotected direct contact in the course of moving around and being attended to medically at the hospital or in the case of a diseased patient during the process of moving to/preparation at mortuary. A typical example of secondary transmission from corpse of infected person to another person that led to infection was the case of a mortician in Germany [9]. These exposed persons then carry the Lassa fever virus back into the community where the cycle of transmission continues with direct unprotected contact; person-to-person or with infected body fluids.

Building early warning indicators and rapid response to adequately prevent or respond to Lassa fever medical care needs at the hospitals, scaling up access to medical supplies and vaccines stockpile are needed in preparedness and during potential epidemics. The following are usually supplied to Local Government Areas (LGAs) at risk in Nigeria [1, 17]. Medicine and disinfectants (ribavirin injection, ribavirin tablet (PEP), medicine for supportive care, ringers lactate, metronidazole (flagyl), oral dehydration salts, bleach). Personal protective and biosafety measures (boots, gloves (thick, thin), outer gown, plastic apron, mask, head cover, protective eye wear, bed nets, etc) best practice as well as equipment (sprayers, plastic sheets for mattress and barriers, water proof mattresses, front lamp, kerosene lamp, body bags, buckets and containers, electric generator) should be adhered at all levels and all times. Public laboratory supplies (needles (different sizes), syringes, tubes (vacutainers) for blood collection, antiseptics) now arises on what quantity of these supplies will be needed during an epidemic so as not to undersupply or overtly over-supply for both extremes are not optimum in resource mobilization and use.

It is important to note that in effectively tackling this scourge, test kits and laboratory analysis to confirm suspected cases as soon as possible need to be readily accessible. Lassa virus and other emerging viral diseases detection and confirmation requires Biosafety level 4 (BSL-4) laboratories across the world, but very few exist in Africa [13]. Some African countries do not even have any. Nigeria has five Lassa fever diagnosis laboratories but it seems only the Irrua Specialist Hospital is fully functional as most suspected cases are sent there for laboratory confirmation [30]. Though knowledge on Lassa fever in Nigeria is high among medical practitioners, low access to affordable and simple tests for timely distinguishing and confirming the disease in the region is observed [31]. These further prolong the time between suspecting a case and confirming it for Lassa fever and its attendant consequences on the disease outbreak and control efforts.

### Accelerating Research and development (R&D) for novel diagnostic tools, drugs and safe vaccines

It is alarming that the true incidence of Lassa fever is unknown as quoted incidences are extrapolations from 1980s studies. Thus the Lassa fever research field is in dire need of more accurate and recent studies on disease incidence, geographic distribution and virus seroprevalence [16, 30]. Rivabirin is currently the recommended medication for Lassa fever [2, 9, 32, 33]. The intravenous form is more efficacious than the oral form [33]. As the race towards Lassa fever vaccine development continues, WHO has put forward its thoughts towards the preferred vaccination measure against a reactive outbreak emergency response [16]. Diagnosis serves as the first step in early disease detection, surveillance and response thus more reliable and effective diagnostic test assays with capacity to detect the five strains of Lassa fever virus are needed. This comes handy as one considers the choices for primers of geographic region specific Lassa fever virus strains.

Accelerating health systems strengthening and rebuilding transformations in affected West Ebola outbreak countries is important through improving ISDR system and scaling up access to routine immunization programs; while leveraging on community-based and vulnerable populations’ empowerment and resilience activities in endemic regions to traveller medical information [34, 35]. Likewise, continuously collective partnership and engagement in medical and veterinary best practices is necessary in human and animal care delivery [35]. For example the consensus on experimental Ebola immunization deployment is endorsed by all parties during the West Africa Ebola virus outbreak of 2013/2014 in most affected countries [35]. However, Nigeria showed strong and effective resilience that gave the country a head start and indeed the first African country to curtail the scourge during the period. Investing in developing and implementing local and regional Lassa fever immunization programs and Lassa virus transmission dynamics interruption interventions supported by effective cold chain and follow-up tracking system, contextual communication in establishing trust and confidence with vulnerable communities are all key value-added approaches and strategies in curbing the prevalent Lassa outbreaks scourge and eventual elimination [34–37].

## Conclusion and recommendations

Lassa fever virus outbreak, a poverty-related infectious diseases outbreak remains a public health threat and burden on vulnerable populations in West Africa and Nigeria in particular. Robust and sustainable leadership commitment and investment of all stakeholders and affected communities in Lassa fever outbreaks prevention and containment is crucial and requires strengthening integrated Lassa fever outbreak surveillance quality data gathering to support evidence data sharing, contextual local and regional outbreak early warning alert, preparedness and response systems. Collaborative ‘One Health’ approach operational research is needed in understanding spatio-geographical risk factors patterns, reservoir(s) mapping and phylogenetic in guiding evidence-based, appropriately tailored and timely integrated programs and strategic interventions implementation against the zoonotic disease epidemics and pandemics threats in Nigeria and the sub-Saharan Africa foci terrain. Furthermore, fast-tracking R&D for more sensitive diagnostic tools, safer and effective drugs and vaccine development is imperative in improving contextual community-based immunization decision making policy to effectively outwit Lassa fever outbreak and other emerging pandemics public health emergencies. Moreover, fostering local community to regional re-merging and emerging epidemics and pandemics data sharing and coordinated invasive pathogens epidemiology surveillance and early warning indicators metrics capacity building, monitoring and evaluation is crucial for timely and quality risk communication and operational research. Integrating community “One Health” surveillance and rapid response approach practice against Lassa fever epidemic and other emerging pandemic threats offers new opportunities in understanding human-animal and environment interface and expanding zoonotic diseases public awareness and resilience strategies and mitigation measures in attaining national and global health security.

## Additional file

Additional file 1: Multilingual abstracts in the five official working languages of the United Nations. (PDF 423 kb)
