# Supplementary material for: Re-emerging Lassa fever outbreaks in Nigeria: Re-enforcing “One Health” community surveillance and emergency response practice
Source: Infect Dis Poverty. 2018 Apr 28;7:37. doi: 10.1186/s40249-018-0421-8 (PMC5923006; doi:10.1186/s40249-018-0421-8)
Supplement: Supplementary file 1 — Multilingual abstracts in the five official working languages of the United Nations. (PDF 423 kb) [file 40249_2018_421_MOESM1_ESM.pdf]

Translation of the abstract into the five official working languages of the United Nations

عودة ظهور وتفشي حمى لاسا في نيجيريا: تعزيز 'الصحة الواحدة' وممارسات الإستجابة لحالات الطوارئ والرقابة المجتمعية.

بقلم: إرنست تامبو، أولواسيجون ت. أديتنده ، البروفيسور أ. الابيبي ،

ملخص

قيّمنا تأثير النزاعات، التي صنعها الإنسان وتأثير التغيرات المناخية في توجيه علم الأوبئة، المستند على"الصحة الواحدة" وممارسات الاستجابة لحالات الطوارئ في مكافحة الأوبئة المعاد ظهورها. وتزايد الأدلة حول ظهور وإعادة ظهور الأمراض حيوانية المصدر، بما في ذلك تفشي حمى لاسا، تقريبا في 20 مدينة في نيجيريا، مما أدى إلى 101حالة وفاة و 175 حالة مشتبه بها وحالات مؤكدة منذ شهر آب/أغسطس 2015. ويضم هذا الرقم75 حالة مؤكدة مختبريا، بما في ذلك 90 حالة وفاة، تمثل 120 ٪ من الوفيات المؤكدة مختبريًا. وقد انتقل الوباء للدول المجاورة مثل بنين، حيث تم الإبلاغ عن 23 حالة وفاة من أصل 68 حالة. وتقوم هذه الدراسة بتقييم الاتجاهات الحالية المتعلقة بإعادة ظهور وتفشي حمى لاسا في فهم المجال الجغرافي للمخازن، وعوامل الخطر، ورسم خرائط لأماكن الإصابة بفيروس لاسا ، والثغرات الكامنة و التحديات التي تواجهها الأنظمة الصحية. وقد ظهر أن الإصابة وتفشي حمى لاسا يبلغ ذروته في موسم الجفاف (في الفترة من كانون الثاني / يناير إلى غاية شهر آذار/ مارس) وتنخفض خلال موسم الأمطار (من شهر أيار/ مايو إلى شهر تشرين الثاني / نوفمبر) سنوياً في سيراليون، والسنغال حتى شرق نيجيريا. و وثقنا ندرة البيانات المتعلقة بقوارض (المخازن) - المرتبطة بتفشي حمى لاسا ،وضعف تدابير الوقاية الثقافية والسلوك الإجتماعي الفعال وإدماج اجراءات المراقبة، وضعف ومحدودية المعرفة والوعي المجتمعي وعدم كفاية القدرة على الإستعداد والوصول إلى إدارة الحالات بيسر في الدول المتضررة. وعليه، أصبح وجود استثمار والتزام قيادي إقليمي ودون الإقليمي في مكافحة حمى لاسا أمر ضروري في بناء إدارة للنفايات متكاملة وفعالة وفي إجراءات الصحة النباتية. إن دمج مجتمع "الصحة الواحدة" للمراقبة و الاستجابة السريعة لنهج قائم على الممارسة في مكافحة وباء حمى لاسا، يوفر فرصًا جديدة في فهم التفاعلات القائمة بين الإنسان والحيوان. والإستفادة من مركز إفريقيا للوقاية ومراقبة الأمراض"CDC"، و تقدم المصادر السحابية و أدوات وسائط التواصل الإجتماعي أمر أساسي في تطوير ودمج الأدلة والإتصال للإبلاغ عن المخاطر في الوقت المناسب، ونظم الإبلاغ من أجل تعزيز الكشف المبكر والمراقبة لتفشي حمى لاسا، وتحذيرات الإنذار وتنفيذ الاستجابة الذكية في الأماكن المتضررة.

Translated from English version into Arabic by Free bird and Nema Alaraby, through

TRANSLATORS
WITHOUT BORDERS

尼日利亚再现拉沙热疫情：重新加强“共享健康”社区监测和应急响应措施

Ernest Tambo , Oluwasegun T. Adetunde,   Oluwasogo A. Olalubi,

摘要

我们评估了人为冲突事件和气候变化对“共享健康”流行病学社区监测和应对新发/再现疫情的应急响应措施的影响。近年来，新发和再现人兽共患病例不断增多。2015 年 8 月以来，尼日利亚近 20 个州暴发了拉沙热疫情，导致 101 例死亡病例，175 例疑似和确诊病例。实验室确诊拉沙热病例为 75 例，但实际死亡数（90）是其 1.2 倍。拉沙热疫情已经传播到邻国，例如，贝宁已报道 68 例拉沙热病例，其中 23 例为死亡病例。本研究评估了拉沙热本次暴发的现行趋势，以了解其孳生区域的空间地理分布；风险因素模式和拉沙病毒发病率地图；卫生系统的内在弱点以及面临的挑战。研究结果表明，在塞拉利昂、塞内加尔至尼日利亚东

部，每年拉沙热发病率和流行率的峰值期与该地旱季（1 月至 3 月）相重叠，而在雨季（5 月至 11 月）降低。我们发现，在该病流行国家，啮齿类动物（贮主）相关拉沙热暴发数据缺乏一致性，文化与社会行为有效防控措施的整合不力，社区防控知识薄弱，应对能力不足和接受可支付治疗受限。因此，拉沙热防控迫切需要强有力的（次）区域领导参与和投资，建立综合有效的废物管理和植物病害控制措施；将“共享健康”社区监测与快速响应措施结合来防控拉沙热疫情，为理解人类与动物之间的相互作用提供了新的思路。借助非洲 CDC 的平台作用，以促进云资源和社会媒体工具及解决方案发展为核心，在易感区域整合循证而及时的风险沟通、早发现早监测的报告系统以及预警警报和智能响应措施。

Translated from English version into Chinese by Pei Wang, edited by Jin Chen

CHINESE CENTER FOR DISEASE CONTROL AND PREVENTION
NATIONAL INSTITUTE OF PARASITIC DISEASES

Réémergence des flambées de la fièvre de Lassa au Nigéria : renforcement du suivi communautaire et entraînement à l'intervention en cas d'urgence selon le principe « Un monde, une santé »

Ernest Tambo, Oluwasegun T. Adetunde, Oluwasogo A. Olalubi

Résumé

Nous avons évalué l'effet des conflits humains et du changement climatique lors de notre accompagnement du travail communautaire d'épidémiologie basé sur la connaissance des faits et des entraînements à l'intervention en cas d'urgence, réalisés selon le principe « Un monde, une santé » pour lutter contre les épidémies émergentes ou réémergentes. Il est de plus en plus évident que l'émergence et la réémergence de maladies zoonotiques, dont les récentes flambées de la fièvre de Lassa dans plus de 20 États du Nigéria, ont causé la mort de 101 individus ; 175 cas suspects ou avérés ont été recensés depuis août 2015. Sur les 75 cas confirmés en laboratoire, 90 personnes sont décédées, ce qui correspond à un taux de létalité de 120 % pour les cas confirmés en laboratoire. La flambée a gagné les pays voisins, tels que le Bénin, où 23 personnes ont péri sur les 68 cas recensés. Cette étude évalue les tendances actuelles dans les flambées réémergentes de la fièvre de Lassa afin de comprendre la répartition spatiale/géographique des réservoirs et les variations des facteurs de risque, de cartographier les manifestations du virus et d'identifier les lacunes et les défis rencontrés par les systèmes de prestations médicales. Il apparait que l'endémicité, l'incidence et la prévalence de la fièvre de Lassa atteignent leur pic pendant la saison sèche, de janvier à mars, avant de retomber au cours de la saison humide, de mai à novembre, dans la région qui s'étend entre le Sénégal, la Sierra Leone et l'est du Nigéria. Nous avons documenté un manque de données cohérentes sur les flambées de fièvre de Lassa liées aux rongeurs (réservoirs) dans les pays touchés. Nous y avons également remarqué une quasi-absence des méthodes culturelles et sociales de prévention efficace et une faible intégration des mesures de contrôle, ainsi qu'un manque de connaissances et de sensibilisation au sein des communautés. Enfin, nous avons documenté une capacité de préparation insuffisante et un accès inadéquat à la prise en charge abordable des cas. Il est donc urgent qu'un leadership dynamique, aux niveaux régional et sous-régional, s'engage et s'investisse dans la lutte contre la fièvre de Lassa pour mettre en place des mesures phytosanitaires et de gestion des déchets

intégrées et efficaces. Intégrer, selon le principe « Un monde, une santé », le suivi communautaire et l'entraînement à l'intervention rapide face aux épidémies de la fièvre de Lassa permet de mieux comprendre les interactions entre humains et animaux. Utiliser le potentiel du Centre africain pour le contrôle et la prévention des maladies (Africa CDC) et exploiter les avancées dans l'externalisation en nuage aussi bien que les outils et solutions des médias sociaux est essentiel pour la mise au point et l'intégration des systèmes de signalisation et d'une communication rapide des risques basée sur les faits, visant à renforcer la détection précoce et le suivi des flambées de la fièvre de Lassa, l'alerte d'avertissement et la mise en œuvre de mesures ingénieuses dans les zones vulnérables.

Translated from English version into French by KM Boestad and Gladis Audi, through

TRANSLATORS
WITHOUT BORDERS

Рецидив вспышек лихорадки Ласса в Нигерии: укрепление общественного контроля в рамках исследования One Health («Одно здоровье») и практики экстренного реагирования

Эрнест Тамбо, Олувасегун Т. Адетунде, Олувасого А. Олалуби,

Аннотация

В настоящей работе была произведена оценка воздействия антропогенных конфликтов и влияния климатических изменений в процессе научно обоснованного эпидемиологического исследования на уровне общин One Health («Одно здоровье»), а также практики экстренного реагирования, направленной на борьбу как с возникновением, так и с рецидивом эпидемий. С августа 2015 года появляется больше свидетельств возникновения и рецидива зоонозных болезней, включая недавние вспышки лихорадки Ласса, которые наблюдались почти в 20 штатах Нигерии и привели к 101 смертельному исходу, а также к 175 предполагаемым и подтверждённым случаям заболевания. Из 75 лабораторно подтверждённых случаев заболевания зафиксировано 90 смертельных случаев, что составляет 120% лабораторно подтверждённых летальных исходов. Вспышка распространилась на соседнюю страну Бенин, где наблюдалось 23 смертельных случая среди 68 зарегистрированных инцидентов заболевания. В данном исследовании проведена оценка текущих тенденций рецидивов по вспышкам лихорадки Ласса с учётом пространственно-географических водохранилищ, закономерности факторов риска, картирования частотности вируса Ласса, неустранимых пробелов, а также возрастающих сложностей в системах здравоохранения. Ежегодно в Сьерра-Леоне, Сенегале и Восточной Нигерии пик эндемичной заболеваемости и распространённости лихорадки Ласса приходится на засушливый сезон (с января по март) и снижается во время сезона дождей (с мая по ноябрь). В исследовании были задокументированы: нехватка достоверных данных относительно взаимосвязи вспышек лихорадки Ласса с грызунами и водохранилищами, слабость с точки зрения культуры и

общественно-поведенческой эффективности по внедрению мер профилактики и контроля, слабые или ограниченные знания и осведомлённость на уровне общин, неадекватный потенциал готовности и приемлемого управления доступом в пострадавших странах. Следовательно, для создания комплексного и эффективного управления отходами и внедрения фитосанитарных мер безотлагательно требуется прочная (суб-)региональная приверженность руководства, а также капиталовложения в борьбу с лихорадкой Ласса. Внедрение общинного надзора «Одно здоровье», а также оперативный подход к практике экстренного реагирования по борьбе с эпидемией лихорадки Ласса открывает новые возможности понимания в вопросах взаимодействия между людьми и животными. Максимальное задействование Центра по контролю и профилактике заболеваний (CDC) в Африке, достижения в области облачных технологий, а также использование социальных информационных средств и решений лежат в основе разработки и интеграции научно-доказательных и своевременных оповещений об опасности, а также внедрения в условиях чрезвычайных ситуаций отчётных систем по усилению раннего выявления и надзора, ранних предупреждений и эффективного реагирования при вспышках лихорадки Ласса.

Translated from English version into Russian by Liudmila Tomanek and Svetlana Podkolzina, through

TRANSLATORS WITHOUT BORDERS

Reaparición de brotes de fiebre de Lassa en Nigeria: refuerzo del programa de vigilancia comunitaria y respuesta ante emergencias «Salud Única»

Ernest Tambo , Oluwasegun T. Adetunde, Oluwasogo A. Olalubi,

Resumen

Analizamos el efecto de los conflictos causados por el hombre y del cambio climático sobre el programa comunitario con base empírica de respuesta ante emergencias y epidemiología «Salud Única» contra epidemias reemergentes. Existe una creciente evidencia sobre la aparición y reaparición de enfermedades zoonóticas, incluidos los brotes recientes de fiebre de Lassa en casi 20 estados de Nigeria que han causado 101 muertos y 175 casos sospechosos y confirmados desde agosto de 2015. De los 75 casos confirmados en el laboratorio, se han producido 90 muertes, que representan un 120 % de mortalidad entre los casos confirmados en laboratorio. Los brotes han alcanzado países vecinos como Benín, donde se han producido 23 muertes entre los 68 casos confirmados. Este estudio evalúa la tendencia actual de la reaparición de los brotes de fiebre de Lassa y estudia la distribución espacial y geográfica de los reservorios, el patrón de los factores de riesgo y la proyección de la incidencia del virus de Lassa, las carencias intrínsecas y los retos a los que se enfrentan los sistemas sanitarios. Se demuestra que la incidencia y la prevalencia de los picos de fiebre de Lassa endémicos se solapan con la estación seca (de enero a marzo) y se reducen durante la estación húmeda (de mayo a noviembre) cada año en Sierra Leona, Senegal y el este de Nigeria.

Hemos documentado que existe una escasez de datos sobre la relación entre roedores (reservorios) y los brotes de fiebre de Lassa, un comportamiento social y cultural débil en lo que refiere a la prevención efectiva y la integración de medidas de control, un conocimiento limitado o insuficiente por parte de la comunidad, una capacidad de preparación inadecuada y una falta de acceso asequible al manejo de los casos en los países afectados. Por lo tanto, se necesita con urgencia la inversión y el compromiso de los líderes regionales y subregionales para crear medidas fitosanitarias y una gestión de residuos integral y efectiva. La integración del programa comunitario de vigilancia y respuesta rápida «Salud Única» en la lucha contra la epidemia de fiebre de Lassa ofrece nuevas oportunidades para entender las interacciones entre humanos y animales. Aprovechar el centro de control de enfermedades Africa, los avances en recursos en la nube y las soluciones y herramientas de las redes sociales es básico para desarrollar e integrar comunicaciones de riesgos oportunas con base empírica, y para favorecer la vigilancia y detección a tiempo de nuevos brotes de fiebre de Lassa, las alertas y la implementación de respuestas inteligentes en entornos vulnerables.

Translated from English version into Spanish by Ribcamar and Mamagui4, through

TRANSLATORS
WITHOUT BORDERS
